# Inflammatory Markers may Determine Postoperative Complications in Cases of Peritoneal Carcinomatosis of Ovarian Origin Treated With Cytoreductive Surgery and Hyperthermic Intraperitoneal Chemotherapy

**DOI:** 10.1002/jso.70243

**Published:** 2026-04-01

**Authors:** Donmez Mustafa, Bisgin Tayfun, Manoglu Berke, Sokmen Selman, Sensoy Basoglu Ecem, Boztas Nilay, Ozgul Ozkardesler Sevda

**Affiliations:** ^1^ Department of Anesthesiology Başakşehir Cam and Sakura City Hospital Istanbul Turkey; ^2^ Department of General Surgery Dokuz Eylul University Medical Faculty Izmir Turkey; ^3^ Department of Public Health Provıncıal Health Dırectorate Corum Turkey; ^4^ Department of Anesthesiology Dokuz Eylul University Medical Faculty Izmir Turkey

**Keywords:** cytoreductive surgery (CRS), hyperthermic intraperitoneal chemotherapy (HIPEC), inflammatory markers, postoperative complications

## Abstract

**Background and Objectives:**

This study aimed to evaluate the ability of perioperative inflammatory markers to discriminate postoperative complications within 6 months in patients with ovarian cancer–related peritoneal metastasis undergoing cytoreductive surgery (CRS) and hyperthermic intraperitoneal chemotherapy (HIPEC).

**Materials and Methods:**

This retrospective cohort study included patients with ovarian cancer–related peritoneal metastasis who underwent CRS and HIPEC between January 2011 and August 2022. Data were obtained from the Hospital Information Management System, patient medical records, and anesthesia charts. Systemic immune‐inflammation index (SII), neutrophil–lymphocyte ratio (NLR), and platelet–lymphocyte ratio (PLR) were assessed preoperatively (within 7 days before surgery) and on postoperative days 1, 3, and 5. Postoperative complications occurring within 6 months were graded according to the Clavien–Dindo classification; major complications were defined as Clavien–Dindo grade ≥III. Receiver operating characteristic (ROC) curve analysis was used to evaluate the discriminatory performance of inflammatory markers.

**Results:**

In the preoperative period, AUCs were 0.655 (95% CI: 0.568–0.742) for NLR, 0.655 (95% CI: 0.568–0.742) for PLR, and 0.689 (95% CI: 0.607–0.772) for SII. On POD3, AUCs were 0.638 (0.551–0.726), 0.619 (0.531–0.707), and 0.673 (0.589–0.758), respectively. On POD5, AUCs were 0.724 (0.645–0.803), 0.695 (0.612–0.779), and 0.740 (0.663–0.818), respectively.

**Conclusions:**

Perioperative NLR, PLR, and SII showed measurable discrimination for 6‐month postoperative complications, with numerically higher AUCs on POD5.

**Abbreviations**
CRS: cytoreductive surgery
GIL: gastrointestinal leakage
HIPEC: hyperthermic intraperitoneal chemotherapy
NLR: neutrophil–lymphocyte ratio
PLR: platelet–lymphocyte ratio
SII: systemic immune‐inflammation index

## 1 Introduction

Cytoreductive surgery (CRS) is a complex surgical procedure aimed at removing all visible intra‐abdominal tumor burden and is frequently combined with hyperthermic intraperitoneal chemotherapy (HIPEC) to target residual microscopic disease. In selected patients with ovarian cancer–related peritoneal metastasis, CRS + HIPEC has been associated with improved oncologic outcomes; however, the procedure is highly intricate and carries a substantial risk of postoperative morbidity and occasional life‐threatening complications [1, 2, 3]. Reported complication rates after CRS and HIPEC range from 12% to 60%, influenced by patient‐related factors (e.g., comorbidities, malnutrition), operative variables (e.g., extent of cytoreduction, blood loss), and treatment‐related effects, including the physiologic stress response to major surgery and intraperitoneal chemotherapy [3, 4].

The early postoperative period is characterized by pronounced metabolic and inflammatory changes. Importantly, these inflammatory responses should be interpreted as part of the postoperative physiologic milieu and as potential correlates of adverse outcomes rather than direct causes of complications. In this context, inflammatory markers derived from routine blood counts have attracted attention as practical tools for perioperative risk stratification, reflecting host response and immune–metabolic balance during and after surgery [5, 6]. Among these, the neutrophil–lymphocyte ratio (NLR) and platelet–lymphocyte ratio (PLR) have been explored in relation to postoperative outcomes in CRS + HIPEC and other oncologic surgical settings. The systemic immune‐inflammation index (SII), calculated as neutrophils × platelets/lymphocytes, has also emerged as a composite marker integrating both inflammatory and immune components and has been evaluated in multiple perioperative contexts [5, 6, 7, 8].

Within CRS + HIPEC cohorts, Amroun et al. reported that elevated postoperative NLR values (postoperative days 5 and 7) were associated with infectious complications in patients undergoing CRS and HIPEC for peritoneal metastases of various origins [9].

More broadly, pro‐inflammatory indices have been associated with postoperative complications across major abdominal and oncologic surgeries, supporting the hypothesis that perioperative inflammatory profiles may help identify patients at higher risk of complications, without implying a direct causal relationship [8, 10].

Nevertheless, evidence specifically addressing the discriminatory performance of perioperative NLR, PLR, and SII at defined time points for predicting postoperative complications after CRS + HIPEC in ovarian cancer–related peritoneal metastasis remains limited.

**Figure 1:**
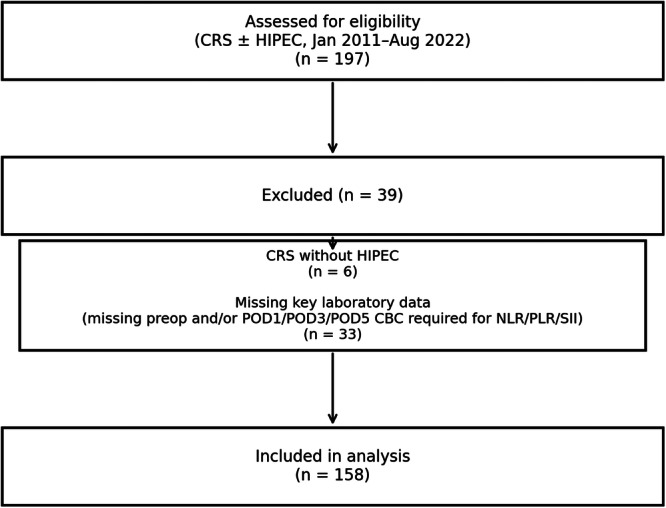
Patient flow diagram (CONSORT‐style).

Therefore, this study aimed to evaluate the ability of preoperative and postoperative (postoperative days 1, 3, and 5) NLR, PLR, and SII values to discriminate postoperative complications occurring within 6 months after CRS and HIPEC in patients with ovarian cancer–related peritoneal metastasis.

**Figure 2:**
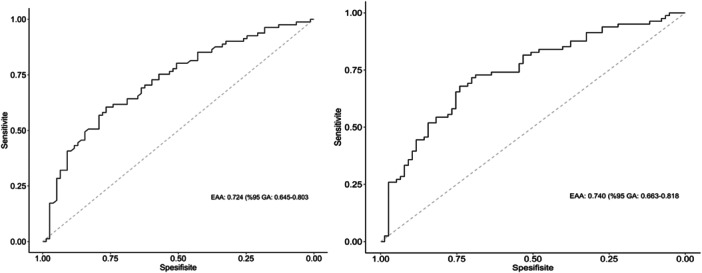
The effectiveness of the NLR and SII values on postoperative day 5 in determining postoperative complications (ROC curve).

## 2 Materials and Methods

### 2.1 Study Design and Patients

All procedures performed in this study were in accordance with the ethical standards of the institutional and national research committee and with the 1964 Helsinki declaration and its later amendments or comparable ethical standards. The study was approved by local ethics committee. All the patients gave their written consent both for the surgery and participation in the study.

This study was a retrospective analysis of prospectively collected data on patients with advanced‐stage epithelial ovarian cancer with peritoneal metastasis who underwent cytoreductive surgery (CRS) combined with hyperthermic intraperitoneal chemotherapy (HIPEC) between January 2011 and August 2022. Patients with primary peritoneal carcinoma were not included.

Patients younger than 18 years of age and those who underwent CRS without HIPEC were excluded from the analysis. Cases were excluded if complete blood count values were missing preoperatively and/or on postoperative days 1, 3, or 5, thereby precluding calculation of NLR, PLR, and SII (*n* = 33). Surgical timing was categorized as upfront (primary) CRS + HIPEC versus CRS + HIPEC after neoadjuvant chemotherapy (interval CRS); in the final cohort (*n* = 158), 41 (25.9%) underwent primary and 117 (74.1%) underwent interval CRS. Synchronous disease was defined as peritoneal metastasis detected at diagnosis or within 6 months, whereas metachronous disease was defined as detection > 6 months after diagnosis (Figure 1).

### 2.2 Surgery

Cytoreductive surgery (CRS) was performed by the same dedicated surgical team for all patients. Preoperatively, mechanical bowel preparation and venous thromboembolism prophylaxis were administered. Intravenous antibiotics (cefuroxime axetil and metronidazole) were given 30 min before the surgical incision and repeated every 3 h during the procedure. The extent of peritoneal disease was evaluated using the Peritoneal Carcinomatosis Index (PCI). The primary objective of CRS was to achieve complete eradication of macroscopic tumor burden.

In cases where complete cytoreduction was deemed unfeasible, palliative interventions such as stoma formation, bypass, or debulking were performed. However, these patients were excluded from the final analysis. Residual disease following CRS was assessed using the “Completeness of Cytoreduction” (CC) classification: CC‐0 (no residual disease), CC‐1 (residual disease < 2.5 mm), CC‐2 (residual disease 2.5 mm–2.5 cm), and CC‐3 (residual disease > 2.5 cm) [1, 2, 3, 4, 5].

### 2.3 Hyperthermic Intraperitoneal Chemotherapy

Following CRS, two inflow drains (positioned in the pelvis and the subhepatic region), two outflow drains, and two thermal probes were placed into the abdominal cavity. HIPEC was administered using a perfusion system (The Belmont® Rapid Infuser RI‐2, MA, USA) via the closed abdominal technique under continuous temperature monitoring [5]

The choice of chemotherapeutic agents was individualized based on multidisciplinary team (MDT) decisions. Cisplatin (75 mg/m², 90 min) and/or mitomycin‐C (10 mg/m², 90 min) were commonly used [4, 6, 7]. Other chemotherapeutic agents, including paclitaxel, doxorubicin, and carboplatin, were occasionally employed in specific clinical contexts [4, 7, 8]. The perfusate was maintained at a constant temperature of 42.5°C throughout the procedure. No concurrent intravenous chemotherapy was administered during HIPEC [3].

### 2.4 Pre/Postoperative Period and Evaluation of Complications

Postoperative complications were graded according to the Clavien–Dindo classification. For severity stratification, complications were categorized as minor (grades I–II) and major (grades ≥III).

Because erythrocyte transfusion and total parenteral nutrition may represent expected supportive care in selected CRS + HIPEC patients, these interventions were reported separately in descriptive analyses. Complications were recorded categorically as either present or absent.

Gastrointestinal leakage (GIL) was defined as encompassing anastomotic leakage, bowel perforation, and/or intestinal fistula. GIL was diagnosed based on clinical, radiological, or intraoperative evidence of extraluminal intestinal content originating from anastomotic sites. Clinical indicators included the presence of intestinal material in surgical or percutaneous drains or at the suture line.

Radiological findings such as perianastomotic fluid collections, extraluminal air, or contrast extravasation into the abdominal cavity were also accepted as diagnostic. Intraoperative confirmation of bowel contents leaking into the peritoneal cavity from an anastomotic defect during reoperation constituted definitive surgical diagnosis.

Perforations unrelated to anastomoses, as well as biliary, urinary, or pancreatic leaks, were not categorized as GIL. Infectious complications were identified based on clinical findings corroborated by radiological imaging and/or positive blood or urine cultures. Postoperative complications were recorded by category; patients could have more than one complication. Clavien–Dindo grade was recorded at the patient level as the maximum grade observed during the follow‐up period.

Mortality was defined as all‐cause death occurring within 6 months after CRS + HIPEC.

Clavien–Dindo grade V was defined as death attributable to a postoperative complication and recorded within the complication‐grading framework. Deaths within 6 months not attributable to a recorded postoperative complication were captured as all‐cause mortality but were not graded as Clavien–Dindo V.

### 2.5 Statistical Analysis

The normality of numerical data was assessed using the Kolmogorov–Smirnov test. Descriptive statistics were presented as mean ± standard deviation for normally distributed variables, and as median with interquartile range (25th–75th percentile) for non‐normally distributed variables. Categorical data were summarized as counts and percentages. Between‐group comparisons of numerical variables were performed using the Student's *t*‐test (for normally distributed data) or the Mann–Whitney *U* test (for non‐normally distributed data).

Categorical variables were compared using the Chi‐square or Fisher's exact test, as appropriate.

Receiver operating characteristic (ROC) curve analysis was performed to evaluate the discriminatory performance of biochemical markers and their derived indices for postoperative complications (yes/no), complication severity (major vs. minor), and 6‐month survival.

As an initial step, marker values were compared between groups using the tests described above. ROC curves were subsequently generated for markers showing significant between‐group differences, and the area under the curve (AUC) was reported with 95% confidence intervals. Optimal cut‐off values were determined using the Youden index, and corresponding sensitivity, specificity, positive predictive value (PPV), and negative predictive value (NPV) were calculated. AUC values were interpreted using commonly cited guideline thresholds (e.g., Hosmer and Lemeshow), where 0.7–0.8 indicates acceptable discrimination, 0.8–0.9 excellent discrimination, and ≥ 0.9 outstanding discrimination [9]. Given the study objective of evaluating discriminatory performance for risk stratification, ROC/AUC was selected as the primary performance metric; findings are interpreted as discrimination/association rather than causation. These guideline ranges are provided for context only; Table 5 presents ROC metrics descriptively, and we did not perform formal statistical comparisons of AUCs across markers or time points

All statistical analyses were conducted using R software (version 4.0.2) with the compareGroups and pROC packages. A two‐tailed *p*‐value < 0.05 was considered statistically significant.

## 3 Results

Our study included 158 consecutive patients who underwent CRS and HIPEC operations for ovarian cancer‐related peritoneal carcinomatosis between January 2011 and August 2022.

When examining the distribution of patients according to their comorbidity status, 55.1% had no comorbidities. Hypertension (*n* = 47) was the most frequently observed comorbidity,

followed by hypothyroidism (*n* = 19).

Among the included patients (n = 158), 41 (25.9%) underwent upfront (primary) CRS + HIPEC and 117 (74.1%) underwent CRS + HIPEC after neoadjuvant chemotherapy (interval CRS) (Table 1).

**Table 1:** Sociodemographic and clinical descriptive characteristics of the patients.

*N* = 158	*n* (%)
Age (mean ± SD)	55.6 ± 11.4
BMI (mean ± SD)	26.2 ± 5.13
Smoking (+)	28 (17.7)
**ASA**	
ASA1	72 (45.6)
ASA2	82 (51.9)
ASA3	4 (2.53)
Comorbidity	71 (44.9)
PCI (mean ± SD)	11.8 ± 5.82
**CC**	
CC 0	117 (74.1)
CC 1	36 (22.8)
CC 2	5 (3.16)
**Operation duration (min) — median (IQR) [range]**	438 (360–494) [210–700]
**HIPEC CT Agent**	
Cisplatin	130 (82.28)
Mitomycin C	4 (2.53)
Cisplatin + Mitomycin C	13 (8.23)
Carboplatin	4 (2.53)
Paclitaxel + Doxorubicin	2 (1.27)
Mitomycin + Doxorubicin	5 (3.16)
**Synchronous/Metachronous**	
Synchronous	62 (39.2)
Metachronous	96 (60.8)
LOS (mean ± SD)	19 ± 17
**Timing of CRS** + **HIPEC (upfront vs. interval after neoadjuvant chemotherapy)**	
Upfront (primary) CRS + HIPEC (no neoadjuvant chemotherapy)	41 (25.9)
CRS + HIPEC after neoadjuvant chemotherapy (interval CRS)	117 (74.1)
Mortality^a^	15 (9.49)
**Clavien Dindo Classification**	
CD1	48 (30.4)
CD2	65 (41.1)
CD3	34 (21.5)
CD4	3 (1.9)
CD5	8 (5.06)

Abbreviations: ASA, American Society of Anesthesiology; BMI, body mass index; CT, chemotherapy; PCI, peritoneal cancer index; SS, cytoreduction score; LOS, length of stay.

a Mortality was defined as all‐cause death occurring within 6 months after surgery. Continuous variables are presented as mean ± SD or median (IQR), as appropriate; categorical variables as *n* (%).

During the first 6 months postoperatively, complications were categorized according to the Clavien–Dindo classification system. Grade 2 complications were generally considered minor; however, cases involving erythrocyte transfusion or total parenteral nutrition were excluded from this category.

Conversely, patients who received antibiotic therapy due to infection were included as having minor complications, comprising 22.7% of the overall cohort. Grades IIIa–V were defined as major complications, accounting for 28.5% of cases. In total, 51.3% of patients developed postoperative complications.

Among these 81 patients, 10 experienced non‐infectious adverse events such as pulmonary embolism, pleural effusion, renal failure, deep vein thrombosis, intracranial hemorrhage, and pneumothorax (Tables 2 and 3).

The ROC analyses of the inflammatory markers at the evaluated time points are presented in Figure 2. Inflammatory marker levels differed between patients with and without postoperative complications across the evaluated perioperative time points, as summarized in Table 4.

**Table 2:** Comparison of patients' Clavien‐Dindo scores with the presence of complications and the distribution of complications.

*N* = 158	*n* (%)
Complications	81 (51.3)
Infectious complications	71 (44.9)
Gastrointestinal leak	12 (7.59)
*Anastomotic leak	5 (3.16)
*Bowel perforation	2 (1.27)
*Intestinal fistula	5 (3.16)
Surgical site infection	46 (29.1)
Urinary tract infection	25 (15.8)
Catheter infection	2 (1.27)
Pneumonia	24 (15.2)
Evisceration	17 (10.8)
Abscess	20 (12.7)
Pleural effusion	17 (10.8)
Renal failure	9 (5.70)
Pulmonary embolism	9 (5.70)
Deep vein thrombosis	5 (3.2)
Other complications (intracranial hemorrhage, pneumothorax)	2 (1.27)

*Note:* Patients could experience more than one complication during the 6‐month follow‐up; therefore, totals across complication categories may exceed the number of patients with complications. Values represent the number of patients in whom each complication category was observed (categories are not mutually exclusive). Clavien–Dindo grade was recorded at the patient level as the maximum (highest) grade observed during follow‐up. Clavien–Dindo grade V denotes death attributable to a postoperative complication; deaths within 6 months not attributable to a recorded postoperative complication were captured as all‐cause mortality but were not graded as Clavien–Dindo V. *Gastrointestinal leak (GIL).

**Table 3:** Patient‐level maximum Clavien–Dindo grade distribution within each complication category (*n* = 158).

Complication category	Total	CD 1	CD 2	CD 3a	CD 3b	CD 4a	CD 4b	CD 5
Gastrointestinal leak (composite)	12	0	0	0	10	0	0	2
Anastomotic leak	5	0	0	0	3	0	0	2
Bowel perforation	2	0	0	0	2	0	0	0
Intestinal fistula	5	0	0	0	5	0	0	0
Surgical site infection	46	0	12	7	17	1	1	8
Catheter infection	2	0	2	0	0	0	0	0
Pneumonia	24	0	6	2	7	2	0	7
Abscess	20	0	5	2	4	1	1	7
Evisceration	17	0	0	3	11	0	0	3
Pleural effusion	17	0	1	2	6	0	1	7
Renal failure	9	0	0	0	0	2	1	6
Pulmonary embolism	9	0	8	1	0	0	0	0
Deepvein thrombosis	5	0	4	1	0	0	0	0
Other complications	2	0	0	1	0	0	0	1

*Note:* Clavien–Dindo grade reflects the patient‐level maximum grade during the 6‐month follow‐up and cannot be attributed to a specific complication event when multiple complications occurred.

**Table 4:** Comparison of inflammatory markers between patients with and without postoperative complications (*n* = 158). Groups are defined by the presence of any postoperative complication within 6 months (yes/no), not by Clavien–Dindo grade.

*N* = 158	No Complications (Median [IQR]) (*n* = 77)	With Complications (Median [IQR]) (*n* = 81)	*p*
Neutrophil (mm³)			
Pre POD1 POD3 POD5	3.40 [2.7, 4.2] 8.9 [6.5, 11.8] 7.3 [5, 8.5] 5.2 [3.8, 6.9]	4.5 [3.2, 5.8] 9.3 [7.6, 12.9] 9.1 [5.9, 13.2] 7.5 [5.7, 10.1]	< **0.001** 0.21 **0.002** **< 0.001**
Platelet (mm³)			
Pre POD1 POD3 POD5	209 [172, 293] 174 [137, 221] 166 [138, 204] 198 [171, 238]	262 [223, 334] 203 [151, 258] 187 [157, 238] 246 [187, 319]	< **0.001** **0.013** **0.006** **0.001**
Lymphocyte (mm³)			
Pre POD1 POD3 POD5	1.8 [1.4, 2.3] 0.6 [0.4, 0.8] 1.1 [0.7, 1.3] 1.3 [0.9, 1.6]	1.6 [1.3, 2.3] 0.7 [0.4, 0.8] 0.9 [0.7, 1.2] 1.1 [0.7, 1.4]	0.223 0.846 0.291 **0.023**
NLR			
Pre POD1 POD3 POD5	1.78 [1.38, 2.6] 13.95 [9.08, 21.41] 6.55 [4.17, 10.21] 4.15 [2.9, 6.2]	2.52 [1.73, 3.89] 14.55 [10, 22.94] 10.76 [6.06, 15.43] 7 [4.62, 11]	< **0.001** 0.791 < **0.001** < **0.001**
PLR			
Pre POD1 POD3 POD5	129 [88.33, 163] 302.5 [189, 450] 166.92 [120, 230] 155.56 [126.25, 214.44]	165 [113.12, 228.33] 370 [220, 480] 197.78 [151.11, 288] 235 [165, 325]	< **0.001** 0.101 **0.01** < **0.001**
SII			
Pre POD1 POD3 POD5	440 [253.87, 608] 2394 [1518.21, 3990] 1064 [707.75, 1605.5] 812.62 [566.67, 1262.47]	696.22 [406, 1169.81] 3595.2 [1848, 5450.33] 1918.12 [1060.2, 3344.1] 1759.85 [938.25, 3052.5]	< **0.001** **0.03** < **0.001** < **0.001**

*Note:* Continuous variables are presented as median [IQR]. Bold values indicate statistically significant differences (*p* < 0.05).

## 4 Discussion

In this ovarian cancer–specific CRS + HIPEC cohort, preoperative and early postoperative inflammatory indices (NLR, PLR, and SII) were higher in patients who developed postoperative complications within 6 months. The clearest separation was observed on POD3–POD5 rather than POD1. ROC analyses were included to describe discriminatory performance for risk stratification and are reported descriptively (Table 5). Reported metrics comprised AUC (95% CI), Youden‐derived cut‐offs, and associated sensitivity, specificity, PPV, and NPV, without qualitative labeling or cross‐comparisons of AUCs across markers or time points. Accordingly, the reported cut‐offs should be considered hypothesis‐generating rather than definitive clinical thresholds and require external validation. We therefore retained the numeric ROC outputs in Table 5 and avoided interpreting AUC magnitudes as “good” or “poor” in the Discussion.

**Table 5:** ROC analysis of inflammatory markers for predicting postoperative complications (cut‐off, AUC, 95% CI, sensitivity, specificity, PPV, NPV).

Parametreler	Cut‐off	AUC	95% CI	Sensitivity	Specificity	PPV	NPV
NLR (Preop)	2.715	0.655	0.568–0.742	0.481	0.818	0.735	0.6
NLR (POD3)	8.236111	0.638	0.551–0.726	0.641	0.649	0.658	0.632
NLR (POD5)	6.286364	0.724	0.645–0.803	0.604	0.766	0.731	0.648
PLR (Preop)	169.972	0.655	0.568–0.742	0.481	0.805	0.722	0.596
PLR (POD3)	174.6886	0.619	0.531–0.707	0.641	0.597	0.626	0.613
PLR (POD5)	201.9805	0.695	0.612–0.779	0.654	0.714	0.706	0.662
SII (Preop)	522.735	0.689	0.607–0.772	0.654	0.675	0.679	0.65
SII (POD3)	1654.312	0.673	0.589–0.758	0.567	0.753	0.707	0.623
SII (POD5)	1232.097	0.740	0.663–0.818	0.679	0.740	0.733	0.686

*Note:* Cut‐off values were selected using the Youden index. AUCs (95% CI) and diagnostic performance measures are reported descriptively.

Abbreviations: AUC, area under the curve; CI, confidence interval; NPV, negative predictive value; NLR, neutrophil‐to‐lymphocyte ratio; PLR, platelet‐to‐lymphocyte ratio; POD, postoperative day; PPV, positive predictive value; SII, systemic immune‐inflammation index.

CRS + HIPEC is associated with a pronounced systemic inflammatory and metabolic response, particularly in the early postoperative period, and postoperative morbidity—especially infectious events—may further amplify circulating inflammatory markers.

Because tumor origin, histology, and disease distribution can influence baseline inflammation and postoperative risk, we restricted the cohort to ovarian cancer to improve clinical homogeneity.

Reported morbidity after CRS + HIPEC varies widely (approximately 12%–55%) across cohorts and definitions [10, 11, 12, 13]. Amroun et al. examined infectious complications within 3 months after CRS + HIPEC in peritoneal carcinomatosis of mixed origins and reported infectious events in 34.2% of patients, with associations involving male sex, higher PCI, high gastrointestinal anastomosis, and erythrocyte transfusion [9].

In our study, complications were assessed over a longer follow‐up window (6 months), capturing both early postoperative and later post‐discharge events, and included both infectious and non‐infectious complications; overall complications occurred in 51.2% of patients and infectious complications in 45.0%. PCI values were of similar magnitude to prior series, and PCI was higher among patients who developed complications, which may reflect greater disease burden and operative complexity.

Major complication rates after CRS + HIPEC have been reported at approximately 14.7%–27% [11, 12]. Van Kooten et al. reported high‐grade postoperative serious adverse events in 26.5% of a cohort predominantly of colorectal origin [12]. Using Clavien–Dindo grading, we defined major morbidity as grade ≥III and observed a major complication rate of 28.5%, broadly consistent with prior reports and plausibly influenced by cohort characteristics and the longer complication ascertainment window. As expected, major complications were associated with prolonged postoperative hospitalization in our cohort.

With respect to SII, Garcia et al. reported ROC analyses of postoperative SII (POD5–POD8) for predicting postoperative complications, with AUCs ranging from 0.602 to 0.748 and day‐specific cut‐offs [11]. In our cohort, SII was measured preoperatively and on POD1, POD3, and POD5; preoperative, POD3, and POD5 SII were higher among patients with complications, whereas POD1 SII was not significantly different. Evidence from other surgical populations also suggests modest and timing‐dependent discrimination: Feng et al. reported discriminatory ability of preoperative SII for postoperative infection in colorectal cancer [13], and Jiao et al. reported modest discrimination for SII measured early after upper abdominal surgery [14]. Taken together, these data support the concept that both surgical context and sampling time point may influence the discriminatory performance of inflammatory indices.

For NLR and PLR, our findings similarly suggest that separation is more apparent from POD3 onward. Amroun et al. observed that statistically significant between‐group differences in postoperative inflammatory parameters emerged from POD3 onward in patients with infectious complications [9]. Fernandez et al. likewise reported higher NLR values on POD5 and POD7 among patients with early postoperative infectious complications [15]. In our cohort, NLR and PLR differed preoperatively and on POD3 and POD5, while POD1 did not differ significantly; lymphocyte counts were numerically lower on POD5 among patients with major complications, consistent with a more pronounced postoperative inflammatory/stress response. Variability across studies likely reflects differences in populations, complication ascertainment, and follow‐up windows.

Hung et al. reported moderate discrimination of preoperative NLR for early mortality after CRS + HIPEC (AUC 0.75). Using an NLR cut‐off of 4.4, they also reported differences in length of stay, major complications, and early mortality between high versus low NLR groups [16].

In our analysis, ROC‐derived cut‐offs were estimated for overall complications and were not intended as thresholds for major morbidity.

Length of stay findings were directionally consistent with prior reports [11, 15]. In our cohort, postoperative length of stay was 11.8 ± 5.16 days in patients without complications and 25.9 ± 21.1 days in those with complications, which may also reflect differences in case complexity, institutional pathways, and local discharge practices.

Six‐month all‐cause mortality in our cohort was 9.5% (15/158). It is important to distinguish all‐cause mortality from Clavien–Dindo grade V, which is reserved for deaths attributable to a postoperative complication; these measures are therefore not necessarily identical.

This study has limitations, including its retrospective single‐center design and potential residual confounding. The lack of a contemporaneous CRS‐only control group prevents isolating the incremental effect of HIPEC (*vs.* CRS itself) on the postoperative inflammatory response. ROC‐derived cut‐offs are hypothesis‐generating and require external validation in prospective CRS + HIPEC versus CRS‐only cohorts.

## 5 Conclusion

In conclusion, preoperative and early postoperative inflammatory indices (NLR, PLR, and SII) were associated with postoperative complications within 6 months after CRS + HIPEC in ovarian cancer–related peritoneal metastasis. Differences were most consistent on POD3–POD5 rather than POD1, supporting the concept that inflammatory indices become more informative in the early postoperative window. These markers may support perioperative risk stratification and help identify patients who warrant closer monitoring; however, clinical utility and optimal thresholds require external validation and prospective confirmation.

## Funding

The authors have nothing to report.

## Synopsis

This study aimed to evaluate the ability of perioperative inflammatory markers to discriminate postoperative complications within 6 months in patients with ovarian cancer–related peritoneal metastasis undergoing cytoreductive surgery (CRS) and hyperthermic intraperitoneal chemotherapy (HIPEC). In this ovarian cancer–specific CRS+HIPEC cohort, preoperative and early postoperative inflammatory indices (NLR, PLR, and SII) were higher in patients who developed postoperative complications within 6 months.

## Data Availability

The data used in this study were obtained from the Dokuz Eylul University Hospital Information Management System (Probel HBYS). Due to institutional policies and patient privacy regulations, the data are not publicly available but are available from the corresponding author upon reasonable request.
